# A Review on Sources and Pharmacological Aspects of Sakuranetin

**DOI:** 10.3390/nu12020513

**Published:** 2020-02-18

**Authors:** Monika Stompor

**Affiliations:** Institute of Medical Sciences, University of Rzeszów, Warzywna 1a, 35-310 Rzeszów, Poland; monika.stompor@gmail.com; Tel.: +48-17-851-68-80

**Keywords:** sakuranetin, metoxhoxylated flavanones, anticancer and antimicrobial activity

## Abstract

Sakuranetin belongs to the group of methoxylated flavanones. It is widely distributed in *Polyomnia fruticosa* and rice, where it acts as a phytoalexin. Other natural sources of this compound are, among others, grass trees, shrubs, flowering plants, cheery, and some herbal drugs, where it has been found in the form of glycosides (mainly sakuranin). Sakuranetin has antiproliferative activity against human cell lines typical for B16BL6 melanoma, esophageal squamous cell carcinoma (ESCC) and colon cancer (Colo 320). Moreover, sakuranetin shows antiviral activity towards human rhinovirus 3 and influenza B virus and was reported to have antioxidant, antimicrobial, antiinflammatory, antiparasitic, antimutagenic, and antiallergic properties. The aim of this review is to present the current status of knowledge of pro-health properties of sakuranetin.

## 1. Introduction

Flavonoids are natural plant polyphenols. Based on their chemical structures and the type of substituents in the aromatic rings, they are classified into several subclasses, such as flavanones, flavonols, flavones, isoflavones, dihydroflavonols, chalcones, anthocyanidins, and catechins. Among them, the large group comprises natural *O*-metylated flavones, flavanones and chalcones. In the human organism, they exert numerous beneficial health effects. The best known are anticancer, antioxidant, antiinflammatory, antiviral, antidiabetic, antimutagenic and antimicrobial ones [1,2,3,4,5,6]. Some of these compounds were also characterised as exerting beneficial physiological effects. Among all the flavonoids, sakuranetin is one of the best characterized plant natural product, and one of the most studied among phenolic compounds. In plants, it is present either in the glycosylated form, named sakuranin, or as the aglycone.

**Scheme 1:**
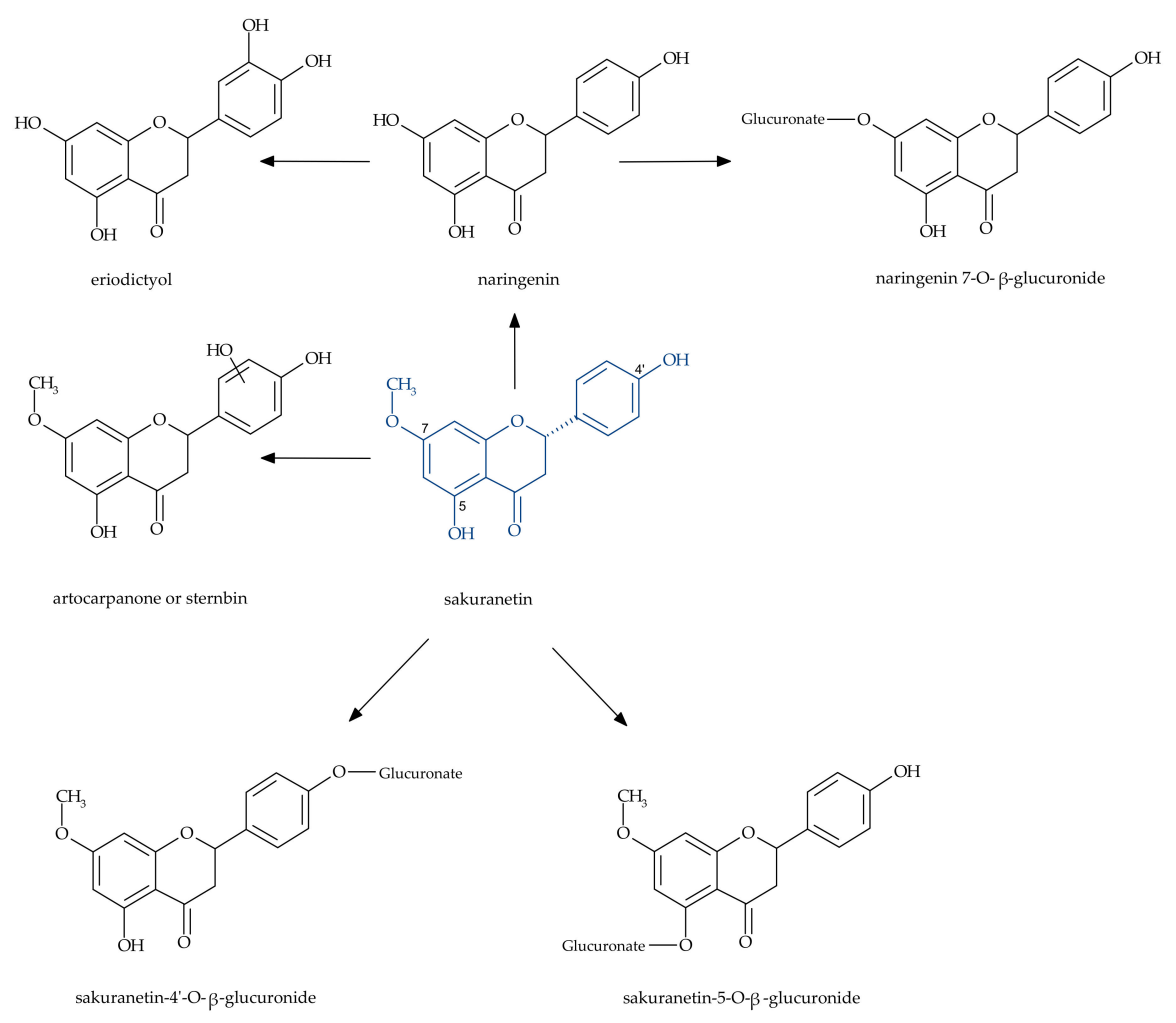
Proposed metabolic pathways of sakuranetin in human liver microsomes [40].

In terms of the chemical structure sakuranetin, chemically named as 4′,5-dihydroxy-7-methoxyflavanone (Scheme 1), it has a molecular weight of 286.27 (C_16_H_14_O_5_) and consist of two fused rings, A and C, and a phenyl ring B, which is attached to the C ring at the C-2 position. This flavanone is characterized by the absence of a double bond between C2-C3 in the C ring, and also by the presence of a 5-hydroxy-7-methoxy substitution pattern in the A ring and a single 4′-hydroxyl group in ring B. Sakuranetin is the *O*-methylated derivative of the best known citrus flavanone naringenin.

In plants, sakuranetin is produced in response to stress and infections [7]. 7-*O*-Methyltransferase, an enzyme that catalyzes the synthesis of sakuranetin, can be activated by ultraviolet light or by infection with *Oryza sativa* [8,9,10]. The key enzyme in the phenylpropanoid pathway is phenylalanine ammonia lyase (PAL), which is directly involved in the synthesis of flavonoid-type phytoalexins, including sakuranetin and naringenin [11]. According to Tomogami’s study, the amino acid conjugates of jasmonic acid are found to elicit the production of the flavonoid phytoalexin sakuranetin in rice leaves [12].

By means of chemical methods, sakuranetin may be synthesized by selective *O*-acetylation of dihydroquercetin using acetic anhydride [13]. It has also been synthesized from glucose in *Escherichia coli* cells through an engineered tyrosine biosynthesis pathway, as described by Kim et al. [14].

**Figure 1:**
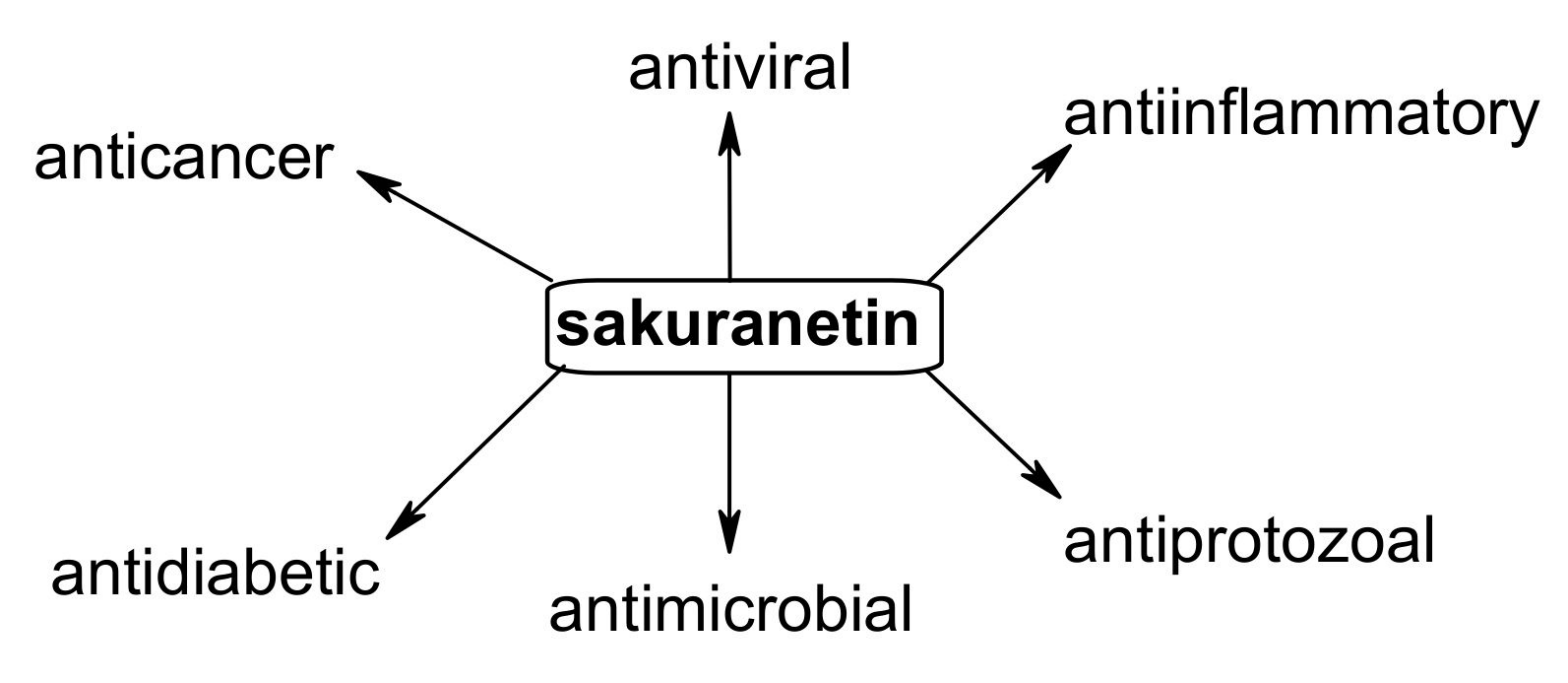
Biological activity of sakuranetin.

Because the mechanisms by which sakuranetin exerts these health-beneficial effects (Figure 1) are not entirely known, the present review focuses on the health-promoting effects of sakuranetin to promote its application in further biomedical studies.

## 2. Sources of Sakuranetin

Sakuranetin is one of the most important natural plant flavonoids (Table 1). Its glycoside, named sakuranin, was isolated for the first time by Asahina et al. from the bark of *Prunus pseudo-cerasus* [15]. The aglycone sakuranetin was first obtained from the bark of *Prunus puddum* [16]. Another report revealed that sakuranetin was first described in 1908 in the cortex of the cherry tree bark (*Prunus* spp.) as the aglycone of sakuranin [17]. According to the literature, sakuranetin was shown to be the main flavonoid found in the species *Baccharis retusa* (family Asteraceae), a plant in southern Brazil [18] from which it was isolated and characterized.

Moreover, the presence of sakuranetin in some varieties of *R. nigrum* may be correlated with their resistance to powdery mildew, whereas its absence may be correlated with their susceptibility to this pathogen. The occurrence of sakuranetin on the surface of leaves of *Ribes nigrum* L. is seasonal and associated with their glands and microflora [19]. Ghisalberti et al. isolated sakuranetin from propolis collected in Western Australia, where it was the major constituent, next to pinostrobin, xanthorrhoeol and pterostilbene [20]. It was also isolated by Agrwal et al. from the methanol extract of the rhizomes of *Iris milesii* [21]. In addition, the compound was isolated by Liu et al. from the leaves and stems of *Eriodictyon californicum* [22] and identified in the leaves of *Hyptis salzmanii* [23]. Methylated flavonoids, including sakuranetin, were isolated from the plants *Teucrium stocksianum* (Labiatae) [24].

Melo et al. noted that sakuneretin is one of the most important compounds involved in the antitumoral activity of *Viscum album* L. (Santalaceae), which is used in complementary medicine fors cancer treatment [25]. Furthermore, sakuranetin was also found in the wood of *Bonnetia dinizii* (Guttiferae) [26], in the bud exudate from *P. sieboldii* (Section Leuce) [27], and in the chloroform-methanol (1:1) extract of *Dodonaea viscosa* (L.) Jacq. (Sapindaceae) [28]. In turn, Aires et al. [29] conducted a study in which they determined the profile and content of phenolic compounds extracted from sweet-cherry (*Prunus avium* L.) stems through a conventional (70 °C, 20 min) and ultrasound-assisted (40 kHz, room temperature, 20 min) extraction. Their results indicate that sweet-cherry stems, except for high content of sakuranetin, also contain considerable amounts of other polyphenolic compounds, including ferulic acid, *p*-coumaric acid, *p*-coumaroylquinic acid, chlorogenic acid and its isomer neochlorogenic acid, which are well known antioxidants.

Sakuranetin was also isolated from the aerial parts of *Dodonaea viscosa* by Zhang et al. [30]. The authors noted that it promoted adipocyte differentiation as characterized by increased triglyceride levels in 3T3L1 cells. Additionally, it was found in the allergy-preventive extract of resins from *Xanthorrhoea hastilis* [31].

Earlier, sakuranetin was also found in *Eupatorium havanense* [32]. Furthermore, De Pascual et al. isolated it from the hexane extract of *Artemisia campestris subsp. glutinosa* [33]. Liang et al. [34] isolated sakuranetin from methanolic extract of powdered stem bark of *Daphne aurantiaca*, the common evergreen shrub native to Yunnan, and Sichuan provinces in China. As a result of the extraction of 6.5 kg of the powder and the subsequent chromatographic purification, 50 mg of pure sakuranetin was isolated. Sakuranetin was also the main compound obtained by purification of 80% ethanol:water extract of *Dicerothamnus rhinocerotis* [35].

Its presence has been confirmed in many other plant species, including *Artemisia campestris*, *Boesenbergia pandurata*, *Baccharis* spp., *Bertula* spp., *Juglans* spp. and *Rhus* spp. Due to their health-promoting effects, these plants were used in folk medicine in the form of herbal supplements, for the treatment of diabetes, inflammatory diseases, allergies and cancer. Furthermore, sakuranetin is a phytochemical abundantly present in many plant extracts [36,37] and the honey of different floral and geographic origins [38] well known for their various biological activities. According to the latest reports, the content of sakuranetin in linden honey was the highest among seven types of honey (Table 2) [38].

## 3. Metabolism of Sakuranetin in the Human Body

The biovailability of flavonones is limited due to presystemic elimination (both in the intestine and the liver). It can be influenced as well by concomitantly taken drugs and other biologically active compounds supplied in daily diet. For this reason, the metabolic pathways of biologically active natural dietary components, including polyphenolic compounds, are being studied [39]. Since flavonoids are unstable compounds, it is likely that the observed effects are related to their degradation products (for example, microbial degradation in the gut or by intestine and colon enzymes) rather than the parent compounds. Many of the flavonoid metabolites have a wide spectrum of biological activity. Therefore, the metabolites of sakuranetin may be also responsible for its therapeutic effects.

The major metabolic pathways of sakuranetin in humans include B-ring hydroxylation, 5-*O*-demethylation, and conjugation with glutathione or glucuronic acid. The phase I metabolites have been identified as naringenin and eriodictyol. Sakuranetin was also found to be a UDP-glucuronosyltransferases (UGT) 1A9 inhibitor, whereas it induced transactivation of the human pregnane X receptor-mediated cytochrome P450 (CYP) 3A4 gene [40].

Benkowić et al. characterized kinetic parameters of *O*-demethylations and aromatic hydroxylations to which selective flavonoid aglycones are susceptible [41]. In this study, sakuranetin underwent sequential biotransformation, starting from demethylation to naringenin, then aromatic hydroxylation to eriodictyol. At the same time, a minor product of direct aromatic hydroxylation of sakuranetin was formed, i.e., 5,3′,4′-trihydroxy-7-methoxyflavanone. Both metabolic pathways were observed in incubations with human liver microsomes and the catalytic effect of sequential biotransformation was 10 times stronger ((0.020 ± 0.005) × 10^6^ M^−1^ min^−1^) compared to hydroxylation at the C-3′ position ((0.0022 ± 0.0002) × 10^6^ M^−1^ min^−1^). Interestingly, the only minor reaction was directly linked with the cytochromes P450 used in this study, i.e., CYP3A4 and CYP1A2, for which catalytic effectiveness constants were (0.06 ± 0.03) × 10^6^ M^−1^ min^−1^ and (0.7 ± 0.4) × 10^6^ M^−1^ min^−1^, respectively.

Ibrahim et al. described the metabolism of sakuranetin by *Cunninghamella elegans* NRRL 1392, which led to naringenin and naringenin-4′-sulfate [42]. However, to date, there have been no reports of the microbial biotransformation of this substance, which could help to understand its biological activity.

Additionally, Katsumata et al. [43] reported that the fungus causing the rice blast disease—*Pyricularia oryzae* (syn. *Magnaporthe oryzae*) metabolized sakuranetin to sternbin and naringenin, which have a lower antifungal activity than the substrate.

According to the literature, a wide range of biological activities have been ascribed to naringenin and eriodictyol, which showed, among others, anticancer, antioxidant, antidiabetic, cytoprotective, and anti-inflammatory properties [44,45,46]. Some studies confirmed the pro-health effects of the derivatives with free hydroxyl groups (e.g., eriodictyol), in contrast to their esters with glucuronic acid [47]. Similarly, the biological properties of naringenin metabolites were often different from those of the aglycone (naringenin). Therefore, it is of paramount importance to test compounds obtained in transformation under physiological conditions, e.g., using microorganisms or in vitro experimental models.

## 4. Biological Potential of Sakuranetin

### 4.1. Anticancer Effects

Methoxyflavonoids are a group of natural substances that have the ability to control the processes of tumorigenesis of various types of cells [48]. Anticancer activity at the molecular level is associated with the modulation of angiogenesis and the influence on cancer cell proliferation and apoptosis [49,50].

Of particular importance is the role of natural phytoestrogens with antitumor activity, which are also an alternative to hormone therapy. They are effective in alleviating menopausal symptoms in women [51]. The results of the studies indicate the importance of phytoestrogens—which contain a methoxyl group in the aromatic ring A—in inhibiting the development of estrogen-dependent breast, ovarian or prostate cancer cells [52,53]. The antioxidative properties of phytohormones and their derivatives are important in preventing the development of tumours [54,55].

Several studies confirmed the correlation between phytochemicals present in daily diet, including flavonoids, and the prevention of lifestyle diseases, including cancer [56]. This group of natural compounds has low adverse effects and systemic toxicity and is safe for human use. Singh et al. noted that the administration of hydroxyl flavones like apigenin, which is the analog of sakuranetin, improved the antioxidant status during carcinogenesis [57]. It was also evidenced that sakuranetin inhibits tumor growth through the apoptosis pathway both in vitro and in vivo. The primary mechanism of its action is the induction of cell death by apoptosis [58]. Park et al. noted that sakuranetin inhibits the growth of human colon carcinoma (HCT-116) cells with an IC_50_ value of 68.8±5.2 μg/mL [59].

Drira and Sakamoto observed that sakuranetin at the concentration of 15 μmol/L had cytotoxic effects on B16BL6 melanoma cells (MTT assay, after 72 h of treatment) [60]. The results indicated that sakuranetin influences the enzymatic process of melanin production (melanogenesis), through the modulation of the signaling pathways in the melanoma cell line. It was proved that sakuranetin inhibits the ERK1/2 and PI3K/AKT signaling pathways, which are involved in the regulation of proliferation, differentiation, and apoptosis, in response to extracellular signals. In this study, the authors proved the upregulating effect of sakuranetin on tyrosinase (Tyr), tyrosinase-related protein 1 (TRP1), and tyrosinase-related protein 2 (TRP2).

Additionally, sakuranetin isolated from *Artemisia dracunculus* was found to have potent effects on the inhibition of cell proliferation in esophageal squamous cell carcinoma (ESCC). This compound induced DNA damage as well as mitochondrial membrane potential loss in esophageal cancer cells [37].

Ugocsai et al. studied the effect of flavonoids and other natural compounds on the reversal of multi-drug resistance (MDR) and apoptosis induction in colon cancer cells [58]. Sakuranetin only had a marginal influence on Rhodamine 123 accumulation in multidrug-resistant Colo 320 human colon cancer cells expressing MDR1/LRP, whereas sakuranin, which is a glucosylated derivative of sakuranetin, was ineffective. Furthermore, there is also some evidence that sakuranetin, being a component of many herbal medicines, may exhance their antiproliferative activity against various human cancer cells, e.g., HT-29 and SGC-7901 [36]. There are also known studies on the relationship between the content of bioactive substances, like flavonoids (including sakuranetin) and free amino acids, and the biological activity of honeys of various origins [38].

### 4.2. Antimicrobial Activity

Compounds of natural origin, due to their high application potential, currently play a significant role in research focused on antimicrobial agents [61,62,63]. With the development of biological methods of treatment, natural substances started to be used as standards or as substrates for the production of more active derivatives [64].

Grecco et al. showed that sakuranetin, which was extracted from twigs of *Baccharis retusa*, could be employed as a tool for designing novel and more efficient antifungal agents [18]. The minimum inhibitory concentration values (MIC) of the isolated compound were determined for pathogenic yeast belonging to the genus *Candida* (six species), *Cryptococcus* (two species/four serotypes) and *S. cerevisiae* BY 4742 (S288c background). The results showed that sakuranetin at a concentration of 0.63 µg/µL inhibited the growth of all the tested *Candida* strains by 98% and 99%, except for *C. albicans*, which was more sensitive to sakuranetin at 0.32 µg/µL (99% of inhibition). The *Cryptococcus* species displayed a similar behavior: *C. neoformans* serotype A (var. grubii) and *C. gatti* (R265) strains in the presence of 0.32 µg/µL of sakuranetin were inhibited by 99% and 97%, respectively. The most sensitive was the strain *C. neoformans* serotype D (JEC21), which showed 98% inhibition with 0.08 µg/µL of sakuranetin concentration.

Previously, the antifungal activity of sakuranetin was also observed for the phytopathogenic strain *Cladosporium* sp. [65] and for the clinical strains *Trichophyton rubrum* and *T. mentagrophytes* [66].

In order to design a new, highly effective compound with a good affinity to the site of action, changes in physical properties of the compound that affect distribution, metabolism and interaction with a particular receptor must be taken into consideration. Thus, strategies to improve the biological impact of a bioactive substance relate to replacements of substituents, stiffening or simplifying the molecule structure, and modifications within the side groups. In the study by Aida et al., sakuranetin was prepared in 75% yield from the main citrus flavanone—naringenin—by treatment with ethereal diazomethane under anhydrous conditions [67]. After acetylating the hydroxy groups, sakuranetin was converted to 7-methoxyapigeninidin by the NaBH_4_ reduction, followed by chloranil dehydrogenation. The results obtained in biological studies suggested that 7-methoxyapigeninidin had a higher antifungal activity than apigeninidin. The results indicated that the presence of the methoxy group at C-7 is important with respect to the antifungal activity against the plant pathogen *Gloeocercospora sorghi*. Apigeninidin, which has no methoxy group, showed only 25% growth inhibition at a two-fold higher concentration (100 ppm).

In turn, Zhang et al. reported sakuranetin as a competitive inhibitor of the β-hydroxyacyl-acyl carrier protein dehydratase from *Helicobacter pylori* (HpFabZ) [68]. The authors suggested that sakuranetin functions as the inhibitor against HpFabZ by competing with the substrate crotonoyl-CoA. It was observed that this activity is strongly correlated with the presence of the methoxyl group in sakuranetin, which does not occur in structurally similar flavonoids quercetin and apigenin, used for comparison. The inhibitory activity of the above-mentioned flavonoids against HpFabZ is as follows: (IC_50_, μM): (*S*)-sakuranetin (2.0 ± 0) > apigenin (11.0 ± 2.5) > quercetin (39.3 ± 2.7). Furthermore, the results obtained using the standard agar dilution method showed that sakuranetin inhibited the growth of *Helicobacter pylori* ATCC 43504 with a minimum inhibitory concentration (MIC) of 92.5 µM.

### 4.3. Antiprotozoal Properties

As it was described, sakuranetin can be helpful for the development of new therapeutic agents to treat Leishmaniasis and Chagas diseases in the future. These are parasitic protozoan diseases that affect the poorest populations in the world, causing high mortality and morbidity.

In the Grecco et al. study, sakuranetin was tested in vitro against *Leishmania spp*. promastigotes and amastigotes and *Trypanosoma cruzi* trypomastigotes and amastigotes [69]. It was confirmed that sakuranetin is active against *Leishmania* (L) *amazonensis*, *Leishmania* (V.) *braziliensis*, *Leishmania* (L) *major*, and *Leishmania* (L) *chagasi* with IC_50_ values in the range of 43–52 µg/mL and against *T. cruzi* trypomastigotes (IC_50_ = 20.17 µg/mL). The results indicated that the presence of both the hydroxyl group at C-4′ and the methoxyl group at C-7 are of paramount importance for antiparasitic activity. Despite the chemical similarity, naringenin, containing three free hydroxyl groups, did not show antiparasitic activity (not active at 150 (promastigotes and trypomastigotes) or 300 μg/mL (amastigotes). Additionally, the methylation of sakuranetin to sakuranetin-4′-methyl ether made the compound inactive against both *Leishmania spp*. and *T. cruzi* (IC_50_ = 265.6 µg/mL against *Leishmania.* (*L.*) *chagasi* promastigotes).

### 4.4. Antiviral Activity

Sakuranetin also has an antiviral activity. Kwon et al. [70] proved that sakuranetin has a strong activity against the influenza B/Lee/40 virus. This activity was shown to be dose- and temperature-dependent. The researchers observed a decrease in the cytopathic effect caused by viral invasion with the 50% inhibitory concentration (IC_50_) of 7.21 µg/mL. The therapeutic index (TI) was over 13.87. The considerable inhibitory effect of sakuranetin on viral RNA synthesis with no visible cytotoxicity was observed at a concentration of 100 µg/mL.

Moreover, Choi reported sakuranetin to be effective against human rhinoviruses HRV3 obtained from ATCC (American Type Culture Collection, Manassas, VA, USA) and propagated in human epithelioid carcinoma cervix (HeLa) cells [71]. Viruses of this type cause the common cold and are associated with the exacerbation of chronic inflammatory respiratory diseases. In the study, Sakuranetin exhibited the excellent antiviral activity of approximately 67% against HRV3 at 100 mg/mL and of approximately 41% at 10 mg/mL.

### 4.5. Antiinflammatory Activity

Since sakuranetin modulates oxidative stress, the NF-κB pathway, and lung function, it may be a candidate for a novel therapeutic agent to prevent and treat acute lung injury (ALI). Bittencourt-Mernak et al. investigated the preventive and therapeutic effects of sakuranetin on lipopolysaccharide (LPS)-induced ALI in mice that were treated with this compound 30 min before or 6 h after instillation of LPS [72]. It was observed that the animals began to show lung alterations 6 h after LPS instillation and these changes persisted until 24 h after LPS administration. Treatment with sakuranetin reduced the neutrophils in the peripheral blood and in the bronchial alveolar lavage. Sakuranetin treatment also reduced macrophage populations, particularly that of M1-like macrophages. In addition, sakuranetin treatment reduced keratinocyte-derived chemokines (IL-8 homolog) and NF-κB levels, collagen fiber formation, MMM-9 and TIMP-1-positive cells, and oxidative stress in lung tissues compared with LPS animals treated with vehicle. Finally, sakuranetin treatment also reduced total protein and the levels of TNF-α and IL-1β in the lung. Mernak et al. have shown a similar effect of sakuranetin which reduced inflammation and collagen deposition in a murine ALI mode [73].

In Kim’s study, the mechanism of the anti-inflammatory activity of sakuranetin was investigated. The study involved using lipopolysaccharide (LPS) and the experimental model with macrophages stimulated with interferon- or LPS [74]. In the cells stimulated with LPS/IFN-γ, sakuranetin inhibited the synthesis of iNOS and COX2. In the case of the single stimulation with LPS, sakuranetin inhibited the secretion of TNF-α, IL-6, and IL-12. The secretion of co-stimulatory molecules CD86 and CD40 was also inhibited. At concentrations of 50 and 100 µM, a decrease in proinflammatory cytokine (TNF-*α*, IL-6, and IL-12) levels was observed as early as after 6 h of incubation.

Sakoda et al. hypothesized that sakuranetin may be a good candidate for the treatment of allergic asthma, caused by inflammation of airways. In vivo sakuranetin treatment in a dose of 20 mg/kg/BALB/c in mice reduced serum IgE levels, lung inflammation (eosinophils, neutrophils, and Th2/Th17 cytokines), and respiratory epithelial mucus production in ovalbumin-sensitized (for 30 days) animals in a murine experimental asthma model. Considering the possible mechanisms, sakuranetin acts by the inhibition of ERK1/2, JNK, p38, and STAT3 activation in lungs. No alterations were found in the livers of treated animals [75].

Santana et al. clarified how sakuranetin treatment (in a dose of 20 mg/kg^−1^/ day; 10 µL intranasal) affects mitogen-activated protein kinases MAPKs and STAT3-SOCS3 pathways in a murine experimental asthma model [76]. Mice were submitted to an asthma ovalbumin-induction protocol and were treated with vehicle, sakuranetin, or dexamethasone. However, sakuranetin did not modify in vitro cell viability in RAW 264.7 and reduced the NO release and gene expression of IL-1β and IL-6 induced by LPS in these cells. These data show that the inhibitory effects of sakuranetin on eosinophilic lung inflammation may be due to the inhibition of Th2 and Th17 cytokines and the inhibition of the MAPK and STAT3 pathways, reinforcing the idea that sakuranetin may be considered a relevant candidate for the treatment of inflammatory allergic airway disease.

The other research team investigated the anti-inflammatory and antioxidant effects of sakuranetin in lung disease using an experimental model of emphysema induced via the instillation of elastase into C57BL6 mice. Sakuranetin in a dose of 20 mg/kg was diluted in 10 µL of a mixture of DMSO: physiological salt solution (1:4) and delivered intranasally. In the sakuranetin-treated emphysematous animals, reductions in lung inflammation, which were associated with attenuated lung parenchymal remodeling and in alveolar destruction, were observed. Sakuranetin treatment reduced lung inflammation and pro-inflammatory cytokine levels (M-CSF, TNF-α, IL-1β, MCP-1 and MIP-2) in lung homogenates [77].

In another study, Yamauchi et al. noted that sakuranetin may be responsible for the antiinflammatory effects of *Pruni Cortex*—the Japanese herbal drug [78]. Sakuranetin, which was present in the ethyl acetate-soluble fraction of the bark extract, significantly inhibited NO induction and inducible nitric oxide synthase (iNOS) expression in rat hepatocytes. Furthermore, this compound decreased the expression of type 1 IL-1 receptor gene and phosphorylation of Akt, also known as protein kinase B, which is regulated by phosphatidylinositol-4,5-bisphosphate 3-kinase (PI3K). Additionally, sakuranetin decreased the phosphorylation of the activator of isoforms of the CCAAT/enhancer-binding protein β (C/EBPβ), which synergistically activates the transcription of the iNOS gene with nuclear factor κB (NF-κB). Therefore, sakuranetin inhibited the co-activating activity of C/EBPβ with NF-κB, leading to the suppression of iNOS gene expression in hepatocytes.

Toledo et al. noted that sakuranetin obtained from *B. retusa*, decreased IgE specific antibodies, eosinophil inflammation, AHR and airway remodelling by reducing oxidative stress, Th2 pro-inflammatory cytokines and chemokines and NF-κB activation in inflammatory cells in an experimental asthma model [79]. Its effects were similar to those observed in animals treated with corticosteroids in the majority of the parameters evaluated.

Zhang et al. studied the impact of sakuranetin and its derivatives such as kaempferol, neosakuranin, sakuranin, sakurenetin-5,4′-di-, 8-D-glucopyranoside, and naringenin, isolated from the methanolic extracts of the stem bark of *Populus davidiana*, on the antiinflammatory activity [80]. The compounds were tested for inhibitory activity against COX-1 and COX-2 enzymes. Sakuranetin showed potent inhibitory activity only against COX-1 (IC_50_ 196.1 µM). The strongest inhibitory effect on both COX-1 and COX-2 was observed for kaempferol (IC_50_ 7.5 µM and 269.2 µM, respectively).

### 4.6. Beneficial Role of Sakuranetin in Alzheimer’s Disease (AD)

Li et al. evaluated the effect of sakuranetin on spatial discrimination in a rat model of cognitive dysfunction exposed to D-galactose, investigated with respect to its effect on malondialdehyde (MDA), superoxide dismutase (SOD) and glutathione peroxidase (GPx) levels, and on the expressions of interleukin-6 (IL-6), tumor necrosis factor-α (TNF-α) and nuclear factor-κB inhibitory factor-α (IκBα) in the hippocampus of rats [81]. The results obtained suggested that sakuranetin may exert protective effects on brain cells through an antioxidation mechanism. Moreover, the improvement in learning and memory impairment by sakurantein may also be related to the inhibition of inflammatory mediators in brain tissue.

### 4.7. Other Effects of Sakuranetin

Furthermore, sakuranetin was also reported to enhance adipogenesis and the insulin sensitivity of 3T3-L1 cells through the upregulation of peroxisome proliferator-activated receptor γ2 (PPARγ2). Saito et al. [82] demonstrated that sakuranetin induces the differentiation of 3T3-L1 preadipocytes, as evidenced by the increased triglyceride accumulation and glycerol-3-phosphate dehydrogenase (GPDH) activity. Moreover, it was observed that sakuranetin stimulated glucose uptake in differentiated 3T3-L1 adipocytes and may sensitize adipocytes to insulin, which suggest that it may contribute to maintaining correct glucose homeostasis in animals.

Hernández et al. proved that sakuranetin inhibits the production of the strongest inflammatory mediators—leukotrienes. It acts as the selective inhibitor of 5-lipoxygenase, the enzyme responsible for their synthesis [83].

Moreover, as it was described, some natural products with methoxyl group and antioxidants might activate or inhibit DNA repair, which may have an effect on inhibiting cancer processes. Double-strand breaks (DSBs) which may be caused, e.g., by reactive oxygen species, disrupt the integrity of DNA in human cells. Failed or improper DSBs repair may lead to genomic instability and, eventually, mutations, cancer, or cell death. Charles et al. noted that sakuranetin in vitro activated the non-homologous end-joining (NHEJ), which is the major pathway used by higher eukaryotic cells to repair these lesions [84].

Flavonoids, including sakuranetin and its derivatives, due to their capability to absorb UV radiation and their well-documented antioxidant activity, classify as anti-aging agents. It was proven that they inhibit the expression of ultraviolet radiation-mediated matrix metalloproteinases (MMPs).

Jung et al. studied the influence of methoxyflavonoids, including sakuranetin, isosakuranetin, homoeriodictyol, genkwanin, chrysoeriol and syringetin on skin photodamage caused by UV-B irradiation [85]. Among all tested substances, the most active was isosakuranetin, which is the isomer of sakuranetin. Isosakuranetin inhibited UV-B-induced phosphorylation of mitogen-activated protein kinase (MAPK) signaling components, ERK1/2, JNK1/2 and p38 proteins. This result suggests that the ERK1/2 kinase pathways likely contribute to the inhibitory effects of isosakuranetin on UV-induced MMP-1 production in human keratinocytes. According to these results, isosakuranetin also prevented UV-B-induced degradation of type-1 collagen in human dermal fibroblast cells. In contrast, sakuranetin was inactive. The other methoxyflavonoids showed no significant inhibition effect on UV-B-induced MMP-1 mRNA expression.

In order to evaluate the bitter-masking potential of sakuranetin, an in vitro study was performed using HEK-293T cells, in which chimeric G-protein α-subunit was expressed [86]. In addition, the cells were transfected with the human bitter receptor, hTAS2R31 which is coupled with G-protein and responsible for bitter taste perception. Sakuranetin at the concentration of 25 µM inhibited the activation of hTAS2R31 by saccharin (1 mM) by over 50% (IC_50_ 5.5 ± 2.5 µM). In order to verify the in vitro results, the activity of 1% ethanolic solution of sakuranetin was confirmed in a bitterness masking test with the participation of four qualified testers. The test was performed in the presence of acesulfame-K, which is a known hTAS2R31 receptor activator. However, the low water-solubility of sakuranetin is a drawback for broader research. Thus, new methods of its functionalization are needed in order to improve its bioavaiability in vivo.

## 5. Conclusions

To summarize, the multidirectional biological effects of sakuranetin are very promising and predispose this compound to further multifaceted research for its use as a drug in many areas of medicine. Although different antimicrobial effects were described, this property is of unknown biological application since the compound also has cytotoxic effects. However, further studies on its pharmacological activity are needed to develop more efficient production methods and new delivery methods. The study should include detailed in vivo tests, further research on anticancer activity involving new cell lines, and investigation of the modulatory role of sakuranetin in biochemical paths (biotransformations). All these may contribute to better understanding the mechanisms of action of natural methoxyflavones (including sakuranetin) and their potential medical use. The aim is to achieve feasible sakuranetin-based clinical formulations.

## Figures and Tables

**Table 1:** The plant sources of sakuranetin.

Plant Name	Part of the Plant	Ref.
*Prunus puddum* (Rosaceae)	bark	[16]
*Prunus* spp. (Rosaceae)	cherry tree bark	[17]
*Baccharis retusa* (Asteraceae)	dried and powdered twigs	[18]
*Ribes nigrum* L. (Grossulariaceae)	leaves	[19]
*Iris milesii* (Iridaceae)	rhizomes	[21]
*Eriodictyon californicum* (Boraginaceae)	leaves	[22]
*Hyptis salzmanii* (Lamiaceae)	leaves	[23]
*Teucrium stocksianum* (Labiatae)	aerial parts	[24]
*Viscum album* L. (Santalaceae)	tinctures	[25]
*Bonnetia dinizii* (Guttiferae)	wood	[26]
*Primula sieboldii* (Primulaceae)	bud exudate	[27]
*Dodonaea viscosa* (L.) Jacq. (Sapindaceae)	-	[28]
*Prunus avium* L. (Rosaceae)	sweet-cherry	[29]
*Dodonaea viscosa* (Sapindaceae)	aerial parts	[30]
*Xanthorrhoea hastilis* (Xanthorrhoeaceae)	dried resin	[31]
*Eupatorium havanense* (Asteraceae)	whole plant	[32]
*Artemisia campestris subsp. glutinosa* (Asteraceae)	-	[33]
*Daphne aurantiaca* (Thymelaeaceae)	stem bark	[34]
*Dicerothamnus rhinoceroses’* (Asteraceae)	dried leaves	[35]

-: no information.

**Table 2:** The content of sakuranetin in honeys [38].

Honey	Acacia	Jujube	Vitex	Linden	Buckwheat	Fennel	Manuka
Sakuranetin [ng/g]	6.49 ± 2.62	7.14 ± 4.55	6.56 ± 4.98	62.2 ± 63.8	17.0 ± 15.0	4.09 ± 2.71	31.6 ± 25.8
